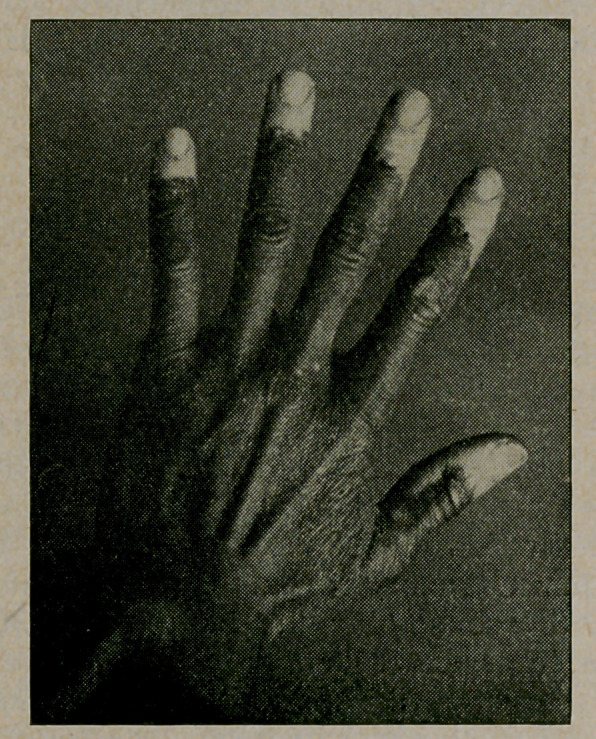# Leocoderma

**Published:** 1915-06

**Authors:** 


**Leocoderma.** L. Griffin of Leesburg, Fla., reports in Med. World, May, 1915, his own case, which is progressive and generalized. He is 35 years old and has no symptoms except itching. The cut, furnished by the World, shows the condition of the fingers.

## Figures and Tables

**Figure f1:**